# Some Technological Properties of Lactic Acid Bacteria Isolated from *Dahi* and *Datshi*, Naturally Fermented Milk Products of Bhutan

**DOI:** 10.3389/fmicb.2017.00116

**Published:** 2017-02-01

**Authors:** H. N. J. Shangpliang, Sharmila Sharma, Ranjita Rai, Jyoti P. Tamang

**Affiliations:** Department of Microbiology, School of Life Sciences, Sikkim UniversityGangtok, India

**Keywords:** technological properties, lactic acid bacteria, *dahi*, *datshi*, naturally fermented milk products

## Abstract

*Dahi* and *datshi* are common naturally fermented milk (NFM) products of Bhutan. Population of lactic acid bacteria (LAB) in *dahi* (pH 3.7) and *datshi* (pH 5.2) was 1.4 × 10^7^ and 3.9 × 10^8^ cfu/ml, respectively. Based on 16S rRNA gene sequencing isolates of LAB from *dahi* and *datshi* were identified as *Enterococcus faecalis, E. faecium, Lactococcus lactis* subsp. *lactis.* LAB strains were tested for some technological properties. All LAB strains except *E. faecalis* CH2:17 caused coagulation of milk at both 30°C for 48 h. Only *E. faecium* DH4:05 strain was resistant to pH 3. No significant difference (*P* > 0.05) of viable counts was observed in MRS broth with and without lysozyme. All LAB strains grew well in 0.3% bile showing their ability to tolerate bile salt. None of the LAB strains showed >70% hydrophobicity. This study, being the first of its microbiological analysis of the NFM of Bhutan, has opened up to an extent of research work that gives a new insight to the products.

## Introduction

**FIGURE 1:**
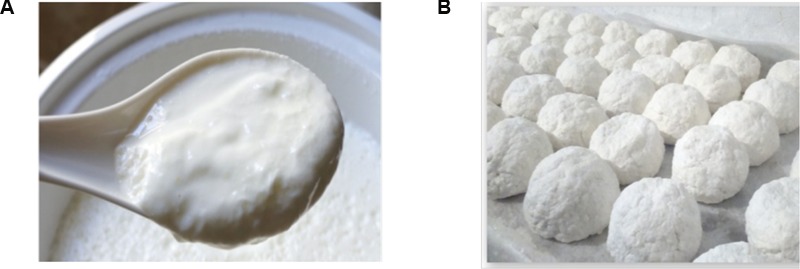
**(A)** *Dahi* and **(B)** *datshi*.

Naturally fermented milk (NFM) products are prepared by the practice of one of the oldest techniques of milk fermentation known as the ‘back-sloping’ method in which a previous batch of a fermented product is used to inoculate the new batch (Josephsen and Jespersen, 2004; Tamang et al., 2016b). NFM products are prepared and consumed daily in Bhutan. Some NFM products of Bhutan are *dahi, datshi, mohi, gheu*, hard-*chhurpi* (*chugo*/*churkam*) and *hitpa*. *Dahi* (**Figure 1A**) is a yogurt-like NFM product of Bhutan, which is traditionally prepared by allowing the boiled milk to undergo spontaneous fermentation at room temperature for 2–3 days with the inoculation of the previous *dahi* sample. *Dahi* is drunk as a refreshing non-alcoholic beverage in Bhutan. *Datshi* (**Figure 1B**) is a cottage cheese like product, which is prepared by churning *dahi* for 10–15 min until a clumping product; butter (locally called *gheu*) is extracted. The butter is collected in another vessel and the buttermilk, locally called *mohi* is then heated for 15–20 min for the curdling of the product, called *datshi*, which is made into round small balls. It is consumed as curry in main meals in Bhutan. Most of these NFM products are occasionally used for religious ceremonies in Bhutan. Some people are economically dependent upon these NFM products where they sell at local markets. Some NFM products of other countries were well studied such as *dahi, misti dahi, shrikhand, chhu, chhurpi, philu* and *somar* of India, Nepal, Pakistan, and Bangladesh (Tamang et al., 2000; Dewan and Tamang, 2006, 2007; Harun-ur-Rashid et al., 2007; Sarkar, 2008; Patil et al., 2010; Tamang, 2010), *kurut* of China (Sun et al., 2010), *aaruul, airag, byasulag, chigee, tarag*, and *khoormog* of Mongolia (Watanabe et al., 2008; Takeda et al., 2011; Oki et al., 2014), *ergo* of Ethiopia, *lben, rayeb, zabady*, and *zeer* of Morocco and Northern African and Middle East countries, *rob* (from camel milk), *biruni, mish* (cow/camel milk) of Sudan, *amasi* (*hodzeko, mukaka wakakora*) of Zimbabwe, *nunu* of Ghana (Akabanda et al., 2013), *filmjölk* and *långfil* of Sweden (Mayo et al., 2010), and *koumiss* or *kumis* or *kumys* or *kymys* of the Caucasian area (Wu et al., 2009). Among species of lactic acid bacteria (LAB), *Lactococcus lactis* subsp. *cremoris*, and *Lc. lactis* subsp. *lactis* are the dominant microbiota along with other mesophilic lactobacilli (*Lactobacillus casei/Lb. paracasei, Lb. fermentum, Lb. helveticus, Lb. plantarum*, and/or *Lb. acidophilus*), *Enterococcus faecium*, species of *Leuconostoc* and *Pediococcus* in NFMs (Tamang et al., 2000, 2016b; Mathara et al., 2004; Dewan and Tamang, 2006, 2007; Patrignani et al., 2006; Watanabe et al., 2008; Wu et al., 2009; Hao et al., 2010; Yu et al., 2011; Akabanda et al., 2013; Oki et al., 2014). Technological properties including probiotics characters have been extensively studied in some NFM products of the world (Patrignani et al., 2006; Dewan and Tamang, 2007; Harun-ur-Rashid et al., 2007; Wu et al., 2009; Tamang et al., 2016a). Till date, there has been no report on the microbiological analysis and technological properties of the NFM from Bhutan, making this research the first of this kind. This paper is aimed to determine some technological properties of the LAB isolates from two popular NFM products of Bhutan- *dahi* and *datshi* such as acidification and coagulation, resistance to low pH, tolerance against bile, lysozyme tolerance and hydrophobicity assay, and also to isolate and identify LAB species by 16S rRNA sequencing.

## Materials and Methods

### Samples

A total number of eight fresh samples of *dahi* (4) and *datshi* (4) were collected from Tabthangbu village, Bhutan in pre-sterilized sampling bags and were transported to the laboratory in an icebox carrier, stored at 4°C and analyzed within a week.

### Microbiological Analysis

Samples (10 ml) were homogenized with sterile physiological saline (90 ml) in a stomacher lab-blender (400, Seward, London, UK) for 1 min, and were serially diluted in the same diluent. LAB were enumerated on MRS agar (M641, HiMedia, Mumbai, India) plates under anaerobic conditions in an anaerobic gas-pack system (LE002, HiMedia, Mumbai, India) and incubated at 30°C for 48–72 h (Dewan and Tamang, 2007). Colonies were selected randomly from the plates which contained less than 10 colonies, according to Leisner et al. (1997). Purity of the isolates was checked by streaking again and sub-culturing on fresh agar plates of the isolation media, followed by microscopic examinations. LAB isolates were preserved at -20°C in MRS broth (M369, HiMedia, Mumbai, India) mixed with 20% (v/v) glycerol.

### Determination of pH

The pH of samples was determined using a pH meter (Crison basic 20, Barcelona, Spain) calibrated with standard buffers.

### Phenotypic Characterization

Cell morphology of all isolates and their motility was determined using a phase contrast microscope (Olympus CH3-BH-PC, Japan). Isolates were Gram-stained and tested for catalase production, and were preliminarily identified based on the phenotypic properties including sugar fermentations, following the methods of Schillinger and Lücke (1987) and Dykes et al. (1994).

### Molecular Identification

#### DNA Extraction

Based on similar sugar fermentation and other phenotypic characteristics criteria, six representative strains of LAB were randomly selected from 44 strains of LAB. Total genomic DNA of six representative strains of LAB was extracted from 2-ml samples of overnight cultures grown in MRS broth at 30°C according to the methods of Martín-Platero et al. (2007). DNA was quantified using fluorometer (Qubitol^®^ 3.0, Fisher Scientific, USA).

#### 16S rRNA Gene Sequencing

The 16S rRNA gene was amplified by PCR mixtures (25 μL) contained approximately 30–50 ng template DNA, 1 μM forward primer 27F and 1 μM reverse primer 1492R (Lane, 1991) using a PCR Master Mix (Promega, Canada) performed under the standard PCR amplification procedure in a SimpliAmp^TM^ Thermal Cycler (Thermo Fisher Scientific, Waltham, MA, USA). The PCR amplicons were checked for their purity on 1% agarose gel electrophoresis in the presence of ethidium bromide (10 mg/mL), which was later analyzed by the Gel Doc System (Ultra-Violet Products Ltd, UK). Sequencing service was outsourced.

#### Phylogenetic Analysis

The BLAST (Basic Phylogenetic Local Alignment Search Tool) program was used for comparing DNA databases for sequence similarities available in the NCBI database. Five different strains/species from each BLAST results were chosen for phylogenetic analysis using Molecular Evolutionary genetics Analysis software (MEGA version 6).

### Technological Properties

#### Activation of LAB Strains

*Enterococcus faecalis* CH1:14, *E. faecalis* CH2:02, *E. faecalis* CH2:17, *E. durans* CH3:03, *Lactococcus lactis* subsp. *cremoris* CH4:01 and *E. faecium* DH4:05, isolated from *dahi* and *datshi*, were grown in MRS broth for 16-24 h at 30°C, and were used for determinations of acidification and coagulation, tolerance against bile, and lysozyme tolerance. Activation of LAB strains for resistance to pH 3 and hydrophobicity were mentioned below.

#### Acidification and Coagulation

Acidification and coagulation ability of LAB strains were assayed by inoculating 10% skim milk (RM1254, HiMedia, Mumbai, India) at 1% level and incubated at 30°C for 72 h. Observation was made for commencement of clotting, followed by pH measurement (Olasupo et al., 2001).

#### Tolerance against Bile

MRS broth containing 0.3% bile was inoculated with active cultures for 4 h (Prasad et al., 1998) and viable cells were enumerated in MRS agar plates after 24 h incubation and growth was recorded.

#### Lysozyme Tolerance

10 mL of MRS broth with lysozyme (MB098-1G, HiMedia, India) and without lysozyme, respectively, was inoculated with 1 mL of both culture suspensions of 10^8^ cfu/ml cell concentration and incubated at 30°C for 24 h and viable cells were enumerated in MRS agar plates after 24 h incubation (Brennan et al., 1986).

#### Resistance to Low pH

Active cultures were harvested by centrifugation and pellets were washed once in phosphate-saline buffer (PBS, pH 7.2), re-suspended in PBS (pH 3) and incubated in MRS agar plates at 30°C for 24 h, and growth was recorded (Prasad et al., 1998).

#### Hydrophobicity Assay

Bacterial affinity to hydrocarbons was determined and results were expressed according to Perez et al. (1998), modified by Tamang et al. (2009) as follows. Fresh cultures were grown in MRS broth at 30°C for 24 h and centrifuged at 8,000 *g* for 5 min. The pellet was washed with 9 ml of Ringer solution (Merck, Germany) and thoroughly mixed. Suspension (1 ml) was taken and the absorbance at 580 nm was measured. Then, 1.5 ml of suspension was mixed with equal volume of n-hexadecane (RM 2238, HiMedia, Mumbai, India) in duplicates and mixed thoroughly. Phases were allowed to separate for 30 min at room temperature, after which aqueous phase was carefully transferred to a new tube and absorbance at 580 nm was measured. The percentage hydrophobicity was expressed as follows:

$$\mathrm{hydrophobicity}\;\%=\left[A_{0}-A/A\right]\times 100,$$

where *A*_0_ and *A* are the absorbance values of the aqueous phase before and after contact with n-hexadecane.

## Results and Discussion

*Dahi* and *datshi* are acidic fermented milk products showing an average pH of 3.7 ± 0.17 and 5.2 ± 0.12, respectively. Isolation of LAB was performed on the classical media i.e., *Lactobacillus* MRS Agar media under anaerobic conditions at 30°C incubation for 48 h. The microbial load of LAB in *dahi* was 1.4 × 10^7^ cfu/ml and in *datshi* was 3.9 × 10^8^ cfu/mL, respectively. A total of 44 LAB isolates were isolated from *dahi* and *datshi* and phenotypically characterized and were randomly grouped into six representative strains based on similar sugar fermentation and other phenotypic characteristics (**Table 1**). These isolates were tentatively identified as *Enterococcus* and *Lactococcus* (**Table 1**).

**Table 1:** Phenotypic characteristics of the lactic acid bacteria (LAB) isolated from *dahi* and *datshi* of Bhutan.

Representative Isolates (no. of grouped strains)	Growth at 45°C	Sugar fermentation											Tentative genera
		Arabinose	Fructose	Galactose	Melibiose	Ribose	Xylose	Raffinose	Aesculin	Melezitose	Salicin	Rhammnose	
^∗^DH4:05 (12)	10/2	7/5	+	+	+	9/3	-	+	+	-	-	-	*Enterococcus*
^∗∗^CH1:14 (3)	+	+	+	+	+	2/1	2/1	+	+	+	+	-	*Enterococcus*
CH2:02 (10)	9/1	+	+	+	-	-	-	+	6/4	5/5	+	-	*Enterococcus*
CH2:17 (4)	2/2	+	-	+	3/1	-	+	2/2	+	+	+	-	*Enterococcus*
CH3:03 (7)	+	+	+	+	3/4	+	+	6/1	+	-	+	+	*Enterococcus*
CH4:01 (8)	6/2	+	-	+	4/4	-	-	-	+	-	-	+	*Lactococcus*

*^∗^DH, denotes isolates from *dahi* samples; ^∗∗^CH, denotes isolates from *datshi* samples. All strains were Gram-positive, catalase negative, coccoids, non-motile and non-sporing; +, all strains positive; -, all strains negative; (../..), number of positive/negative strains. All strains grew at 10 and 15°C. All strains fermented cellobiose, mannose and maltose.*

**FIGURE 2:**
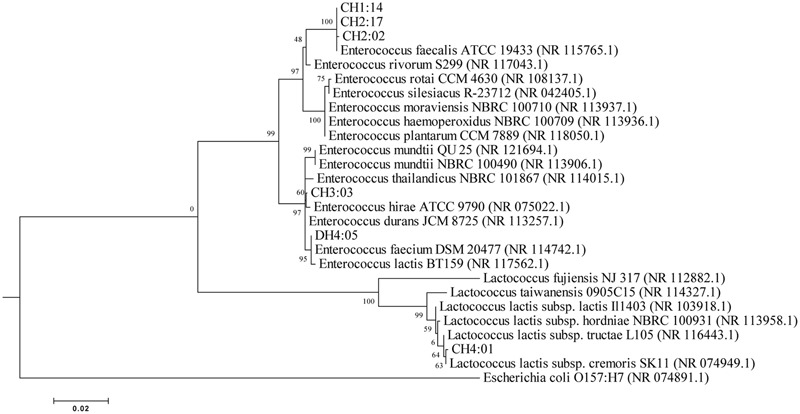
**Phylogenetic tree based upon the Neighbor-Joining of 16S rDNA sequences (*E. coli* 84 to 1437) derived by PCR with the primer 27F and 1492R**.

Total genomic DNA of 6 representative strains of LAB was extracted and amplified and were identified by partial 16S rRNA gene sequencing which were compared to the NCBI database for their phylogenetic relationship by using the software MEGA 6 (**Figure 2**). On the basis of molecular identification, the following species of LAB were identified from *dahi* and *datshi* of Bhutan with percentage similarity of LAB: *E. faecalis* CH1:14 (99%), *E. faecalis* CH2:02 (99%), *E. faecalis* CH2:17 (99%), *E. durans* CH3:03 (99%), *Lactococcus lactis* subsp. *cremoris* CH4:01 (99%), and *E. faecium* DH4:05 (99%; **Table 2**).

**Table 2:** Identification table based on NCBI-BLAST.

Isolates	Length (bp)	Max Score	Query coverage (%)	*E*-value	% Identification	Closest Known Relative (Strain No., GenBank Accession No.)
CH1:14	1406	2591	100	0.0	99	*Enterococcus faecalis* (ATCC 19433, NR 115765.1)
CH2:02	1370	2525	100	0.0	99	*Enterococcus faecalis* (ATCC 19433, NR 115765.1)
CH2:17	1386	2556	100	0.0	99	*Enterococcus faecalis* (ATCC 19433, NR 115765.1)
CH3:03	1384	2536	99	0.0	99	*Enterococcus durans* (JCM 8725, NR 113257.1)
CH4:01	1361	2508	100	0.0	99	*Lactococcus lactis* subsp. *cremoris* (SK11, NR 074949.1)
DH4:05	1378	2542	100	0.0	99	*Enterococcus faecium* (DSM 20477, NR 114742.1)

*Lactococcus lactis* subsp. *lactis, Lc. lactis* subsp. *cremoris, E. faecium, E. faecalis, Leuconostoc mesenteroides* and *Pediococcus* and lactobacilli (*Lactobacillus casei, Lb. fermentum, Lb. helveticus, Lb. plantarum*, and/or *Lb. acidophilus*), were reported from many NFM products of different countries (Tamang et al., 2000; Mathara et al., 2004; Dewan and Tamang, 2006, 2007; Patrignani et al., 2006; Watanabe et al., 2008; Wu et al., 2009; Hao et al., 2010; Yu et al., 2011; Akabanda et al., 2013).

Lactic acid bacteria strains were tested for some technological properties (**Table 3**). All LAB strains except *E. faecalis* CH2:17 caused coagulation of milk at both 30°C for 48 h with a significant drop in pH (**Table 3**). Coagulation of milk by LAB strains reveals their potential as starters or adjunct cultures in the production of NFM of Bhutan. Only *E. faecium* DH4:05 strain showed positive result indicating its resistance to pH 3 in applied method (**Table 3**). Resistance to pH 3 is often u**s**ed *in vitro* assays to determine the resistance to stomach pH (Prasad et al., 1998). Resistances to the lysozyme by all six strains of LAB were evaluated in MRS broth with and without lysosome at 30°C for 24 h (**Table 3**). Lysozyme is capable of lysing bacteria, but it doesn’t impair activities of LAB (Saran et al., 2012). Tolerance against bile was also tested and found that all LAB strains grew well in 0.3% bile showing their ability to tolerate bile salt. The mean intestinal bile concentration is 0.3% (w/v) and the staying time of food in small intestine is suggested to be 4 h (Prasad et al., 1998). The probiotic bacteria survival in the gastrointestinal transit is primordial, and implies in the ability of microorganisms to survive at the stomach acidity and bile, so that they can exert their beneficial effects on the host (Pozza et al., 2011).

**Table 3:** Technological properties of the LAB isolates from *dahi* and *datshi* of Bhutan.

Isolates	pH at Commencement of clotting	Coagulation (hours)		Resistance to pH 3	aaaLysozyme tolerance	bbbBile tolerance	(%) Hydrophobicity
		24	48				
*E. faecium* DH4:05	5.54	-	+	+	+	+	17.53
*E. faecium* CH1:14	5.24	-	+	-	+	+	56.58
*E. faecalis* CH2:02	5.52	-	+	-	+	+	8.91
*E. faecalis* CH2:17	5.50	-	-	-	+	+	5.99
*E. faecium* CH3:03	5.00	+	+	-	+	+	1.3
*Lc. lactis* subsp. *lactis* CH4:01	4.70	+	+	-	+	+	3.02

*Data represent an average of three sets of experiments. +, indicates growth (>10^6^ cfu/ml) of LAB strains; aaano significant difference (*P* > 0.05) of viable LAB counts in MRS broth with and without lysozyme after incubation (30°C/24 h) was considered as a strain resistant to lysozyme.; ^b^MRS broth with 0.3% bile.*

Bacterial affinity to hydrocarbons, such as hexadecane, proved to be a simple method to determine cell surface hydrophobicity (van Loosdrecht et al., 1987). None of the LAB strains showed >70% hydrophobicity (**Table 3**). A percent hydrophobic index greater than 70% was classified as hydrophobic (Nostro et al., 2004). Hence, LAB strains from *dahi* and *datshi* do not show hydrophobic character in the applied method. However, these limited technological properties are not enough to validate the potential probiotic uses of these isolates.

## Conclusion

Based on 16S rRNA gene sequencing isolates of LAB, isolated from *dahi* and *datshi* of Bhutan, were identified as *E. faecalis, E. faecium, Lactococcus lactis* subsp. *lactis* and some strains showed promising technological properties. This is the first report on NFM of Bhutan, which may be used as baseline data for further research on NFM products.

## Author Contributions

HS: Molecular analysis of LAB isolates. SS: Isolation and phenotypic characterization. RR: Determination of technological properties of isolates. JT: Compilation of data and preparation of manuscript.

## Conflict of Interest Statement

The authors declare that the research was conducted in the absence of any commercial or financial relationships that could be construed as a potential conflict of interest.
